# Draft Genome Sequence of *Bacillus farraginis* R-6540^T^ (DSM 16013), a Spore-Forming Bacterium Isolated at Dairy Farms

**DOI:** 10.1128/genomeA.00562-16

**Published:** 2016-06-16

**Authors:** Jie-ping Wang, Bo Liu, Guo-hong Liu, Ci-bin Ge, Rong-feng Xiao, Xue-fang Zheng, Huai Shi

**Affiliations:** Agricultural Bio-Resources Research Institute, Fujian Academy of Agricultural Sciences, Fuzhou, Fujian, China

## Abstract

*Bacillus farraginis* R-6540^T^ is a Gram-positive, aerobic, and spore-forming bacterium with very high intrinsic heat resistance. Here, we report the 5.32-Mb draft genome sequence of *B. farraginis* R-6540^T^, which is the first genome sequence of this species and will promote its fundamental research.

## GENOME ANNOUNCEMENT

The type strain R-6540^T^ (= LMG 22081^T^ = DSM 16013^T^) was isolated from the mixed fodder for cattle at dairy farms and named *Bacillus farraginis* (1). Except for its taxonomic literature, no additional information about *B. farraginis* has been obtained so far. Because there is no available genomic information for *B. farraginis*, its type strain (6540^T^) was selected as one of the research objects in our genome sequencing project for genomic taxonomy and phylogenomics of *Bacillus*-like bacteria. Here, we present the first draft genome sequence of *B. farraginis*.

The genome sequence of *B. farraginis* 6540^T^ was obtained by paired-end sequencing on an Illumina HiSeq 2500 system. One DNA library with an insert size of 464 bp was constructed and sequenced. After filtering of the 0.53 Gb of raw data, 0.50 Gb clean data were obtained, providing approximately 94-fold coverage. The reads were assembled via the SOAPdenovo software version 1.05 (2), using a key parameter K setting at 71. Through the data assembly, 86 scaffolds with a total length of 5,320,788 bp were obtained, and the scaffold *N*_50_ was 145,828 bp. The average length of the scaffolds was 61,870 bp, and the longest and shortest scaffolds were 482,514 bp and 502 bp, respectively. Of the clean reads, 94.16% could be aligned back to the genome, which covered 98.41% of the sequence.

The annotation of the genome was performed using the Glimmer, RNAmmer, and tRNAscan-SE tools (3). A total of 5,597 genes were predicted, including 5,432 coding sequences, 141 tRNAs, and 24 rRNA genes. There were 3,703 and 2,181 genes assigned to the COG and KEGG databases, respectively. The average DNA G+C content was 34.74 %, having some difference with the former value 43.7 mol% (1).

### Nucleotide sequence accession numbers.

This whole-genome shotgun project has been deposited at DDBJ/EMBL/GenBank under the accession number LMCA00000000. The version described in this paper is the first version, LMCA01000000.

## References

[1] Scheldeman P, Rodríguez-Díaz M, Goris J, Pil A, De Clerck E, Herman L, De Vos P, Logan NA, Heyndrickx M 2004 *Bacillus farraginis* sp. nov., *Bacillus fortis* sp. nov. and *Bacillus fordii* sp. nov., isolated at dairy farms. Int J Syst Evol Microbiol 54:1355–1364. doi:10.1099/ijs.0.63095-0. 15280314

[2] Li R, Zhu H, Ruan J, Qian W, Fang X, Shi Z, Li Y, Li S, Shan G, Kristiansen K, Li S, Yang H, Wang J, Wang J 2010 *De novo* assembly of human genomes with massively parallel short read sequencing. Genome Res 20:265–272. doi:10.1101/gr.097261.109. 20019144 PMC2813482

[3] Aziz RK, Bartels D, Best AA, DeJongh M, Disz T, Edwards RA, Formsma K, Gerdes S, Glass EM, Kubal M, Olsen GJ, Olson R, Osterman AL, Overbeek RA, McNeil LK, Paarmann D, Paczian T, Parrello B, Pusch GD, Reich C, Stevens R, Vassieva O, Vonstein V, Wilke A, Zagnitko O 2008 The RAST server: Rapid Annotations using Subsystems Technology. BMC Genomics 9:75. doi:10.1186/1471-2164-9-75. 18261238 PMC2265698

